# Sex pilus specific bacteriophage to drive bacterial population towards antibiotic sensitivity

**DOI:** 10.1038/s41598-019-48483-9

**Published:** 2019-08-30

**Authors:** Joan Colom, Diego Batista, Abiyad Baig, Ying Tang, Siyang Liu, Fangzhong Yuan, Aouatif Belkhiri, Lucas Marcelino, Fernanda Barbosa, Marcela Rubio, Robert Atterbury, Angelo Berchieri, Paul Barrow

**Affiliations:** 1 School of Veterinary Medicine and Science, University of Nottingham, Sutton Bonington, Leicestershire LE12 5RD UK; 2 School of Agricultural and Veterinary Sciences, São Paulo State University (FCAV/UNESP), São Paulo, 14884-900 Brazil

**Keywords:** Bacteriophages, Antimicrobial resistance

## Abstract

Antimicrobial resistance (AMR) is now a major global problem largely resulting from the overuse of antibiotics in humans and livestock. In some AMR bacteria, resistance is encoded by conjugative plasmids expressing sex-pili that can readily spread resistance through bacterial populations. The aim of this study was to use sex pilus-specific (SPS) phage to reduce the carriage of AMR plasmids. Here, we demonstrate that SPS phage can kill AMR *Escherichia coli* and select for AMR plasmid loss *in vitro*. For the first time, we also demonstrate that SPS phage can both prevent the spread of AMR *Salmonella* Enteritidis infection in chickens and shift the bacterial population towards antibiotic sensitivity.

## Introduction

Antimicrobial resistance (AMR) results from both regulated and unregulated use of antibiotics and other chemotherapeutic agents for disease treatment in both human and veterinary medicine, and as growth promoters in livestock rearing^1,2^. Transmission of resistant bacteria is exacerbated by globalisation of the animal and food trades, and increased travel^3^. However, modern medicine relies heavily on their use and they currently remain essential for many medical interventions including cardiac and bowel surgery, neonatal care and cancer treatment. AMR is now seen as one of the most severe global threats to health^4^.

The economic cost of AMR is difficult to judge, but the negative effects include medication costs and reduced Gross Domestic Product (GDP) estimated at between 2–3.5% globally. The yearly cost to the United States (US) health system has been estimated at US $21-$34 billion dollars, and more than 8 million additional days in hospital^4^. It is estimated that by 2050, the global cost could reach $65 trillion and could lead to 10 million deaths per year^4^. Individual patients have already died from untreatable bacterial infections as a result of AMR^5^.

Global antibiotic consumption increased by 36% from 2000 to 2010 with 76% of this in the BRICS countries, which includes Brazil^6^. There are now signs of increasing controls on antibiotic use in many countries where historically there has been none^7^ but the frequency of resistance to key chemotherapeutic antibiotics in pathogens such as *Salmonella enterica* and *Staphylococcus aureus* may nevertheless reach 100% of strains in such countries^8–10^. Transmissible resistance to colistin, one of the last resort antibiotics, has also been reported in humans, pigs and meat in China^11^ and several other countries.

AMR in indicator organisms such as *E*. *coli* in pigs and chickens is common in many countries^9,12^, with transmission from livestock to human, mainly via the food chain^12,13^. The recent O’Neill report highlighted the role of *E*. *coli*, both as a commensal and pathogen, as a significant driver of resistance^4^. Much resistance in this organism is plasmid-mediated and transmissible, including resistance to antimicrobials which 20 years ago were chromosomally located such as quinolones and colistin. In the absence of antibiotic selective pressure, plasmid-mediated resistance is frequently associated with reduced fitness in bacteria^14^.

The World Health Organization (WHO) recently published a list of pathogens prioritised in order of greatest threat to human health and for which new antibiotics are urgently required^15^. This includes carbapenem-resistant and ESBL-producing members of the *Enterobacteriaceae*, especially *E*. *coli* and *Klebsiella pneumoniae*^15^. The WHO has also called for a comprehensive strategy to address AMR^4,16^, with tighter national regulation on antimicrobial use. However, even with such controls the problem of resistance will remain acute for decades. Additional recommended approaches to countering AMR include improvements in diagnosis, the identification of new drugs, and the development of alternative approaches to tackle the problem, including the use of phages^16^.

Under carefully controlled conditions, lytic phages have been shown to be highly effective in controlling or preventing a variety of infections^17,18^ including *E*. *coli* septicaemia in mice, chicken and colostrum-deprived calves^19–21^ and intestinal colonisation by *Salmonella*^22^ and *Campylobacter*^23,24^. Most phages attach to surface receptors which are also virulence determinants such as capsules^19^ or lipopolysaccharide^20^. Some phages are known to attach to a range of self-transmissible plasmid-mediated sex pili, which differ in their specificity according to the plasmid incompatibility (Inc) group^25,26^. Sex pilus-specific (SPS) phages, particularly incompatibility group F-specific phages, have been used in bacterial genetics for the identification of bacterial cells possessing the F factor and IncF related plasmids^27^. Interestingly, the level of pili expression varies depending on the plasmid. Some of the plasmids in the F group are classified as derepressed and constitutively express F pili in all conditions^28^. However, other natural IncF plasmids are more repressed and will only express the conjugative pili at low levels and under certain conditions^29^. The application of SPS phages should not only kill bacteria harbouring such plasmids, but any phage-resistant mutants should have lost the plasmid. This could result in bacteria becoming antibiotic sensitive, if the plasmid harboured AMR genes. We have used a model system comprising the F*lac* plasmid in a laboratory *E*. *coli* strain, and the SPS RNA phage MS2^30^ to study plasmid loss *in vitro*. We also transferred the plasmid to a *Salmonella enterica* ser. Enteritidis strain to study phage activity and plasmid loss *in vivo* in a chicken model of infection. Although the F plasmid is unusual as a completely de-repressed plasmid it acts as a good model for this approach to addressing AMR particularly since many wild-type plasmids also show varying degrees of de-repression^31^.

## Results

### The F-specific phage MS2 select for loss of F-like plasmids

**Figure 1:**
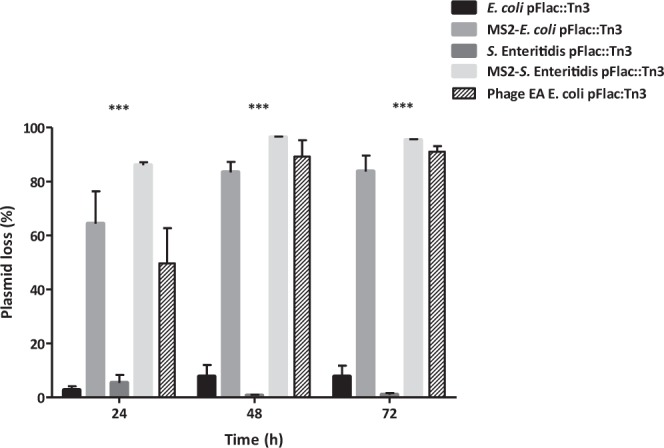
Phage MS2 and phage EA influence on de-repressed plasmid pF*lac*::Tn*3* populations over time. Bars show mean values of three independent experiments ± standard error. Significance was calculated using the Two-way ANOVA with Bonferroni post-tests. ***P < 0.001.

The effect of the SPS phage MS2 and the LPS-specific phage EA on plasmid population dynamics was initially tested with a highly self-transmissible F*lac*-plasmid model using an *E. coli* J62 carrying the F*lac*-plasmid tagged with Tn*3* (Amp^R^). For that, MS2 was added to a late mid-exponential culture of this strain at a multiplicity of infection (MOI) of 10 and incubated for 24 h. After incubation, 64 ± 12.0% of the bacterial population had lost the plasmid in contrast to a bacterial culture incubated without phage where the loss was 2.5 ± 1.5% (Fig. 1) (P < 0.001). Similar results were observed with the LPS phage EA, reducing a 49.7 ± 12.9% the presence of plasmid (Fig. 1, P < 0.001). Because it has been shown that the presence of large transmissible plasmids can sometimes reduce the growth rate of bacteria in the absence of the environmental conditions that select for the plasmid maintenance^14,32^. As such, continued passage in the presence of MS2 was expected to increase the percentage of the plasmid-negative derivatives. The results showed that after 48 and 72 h plasmid loss increased to 83 ± 3.9% under the selective pressure of phage MS2 and remained very low when the phage was absent (7.5 ± 4.3%). Very similar results were observed when the same experiment was performed with phage EA, where plasmid carriage was reduced by up to 91 ± 2.1 (Fig. 1, P < 0.001). Despite these similarities, only the SPS-phage MS2 was able to reduce plasmid carriage in a *S*. Enteritidis P125109 F*lac*::Tn*3* (Fig. 1, P < 0.001). This is a consequence of the type of receptor used by the phage. A LPS phage will be restricted to strains closely related to each other while the SPS-phage, using the pilus encoded by the plasmid will not have restrictions in strain specificity. This highlights the advantage of using SPS phage over classic phage using LPS as a receptor to attach to the bacterial host.

### Characterisation of phage-resistant bacteria

Phage MS2 activity on the highly transmissible F plasmid derivative F*lac*::Tn*3* resulted in an increased fitness of phage-resistant, plasmid-free derivatives above that of phage-resistant, plasmid-containing mutants. Two randomly-selected phage-resistant *E*. *coli* J62 F*lac*::Tn*3* derivatives (R3 and R4) were characterised further. In both mutants MS2 adsorption was almost non-existant, being significantly reduced an 84.6% (R3) and 92.4% (R4) after 14 min compared to the parental F*lac*::Tn*3* (Table 1). Neither of these mutants could transfer the plasmid and ampicillin resistance to *E*. *coli* 711Rif^R^ (Table 1). The new phenotype may be explained by substantial changes in sex-pilus structure or expression. Full sequencing of the R4 mutant revealed a mis-sense mutation in the *traJ* gene (Supplementary Data 1). It is known that the *traJ* gene is a positive regulator involved in the expression of the transfer genes in plasmids^33^. Therefore, a missense mutation in it would lead to a reduced or inexistent conjugation ability as reflected by the results obtained in this section.

**Table 1:** Plasmid transfer rate of MS2 phage-resistant Flac::Tn3 *E. coli* strains.

Plasmid	MS2 sensitivity	Transfer rate (%)	MS2 adsorption (%)*	Reduction in adsorption (%)
F*lac*::Tn*3* (parent)	YES	95.7 ± 3.5	86.8 ± 5.1	0
F*lac*::Tn*3* R3	NO	0.0 ± 0.0	13.4 ± 10.1	84.6
F*lac*::Tn*3* R4	NO	0.0 ± 0.0	6.6 ± 6.6	92.4

*Adsorption after 14 minutes in contact with the strains carrying plasmids. The values presented are the mean of three independent experiments (n = 3) ± standard error.

### Phage MS2 drives *Salmonella* Enteritidis towards plasmid loss and antibiotic sensitivity during infection of chickens

**Figure 2:**
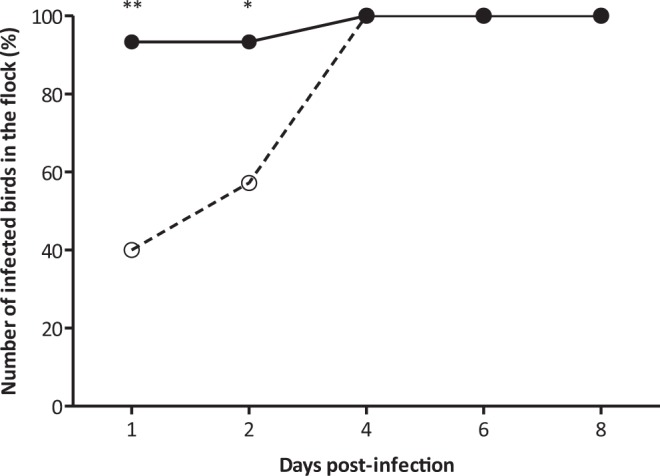
*S*. Enteritidis P125109 F*lac*::Tn*3* spread in whole chicken flock. Phage MS2 treated group (○) and control animals (•). Values represent mean proportion of infected birds in the flock (n = 15). Significance was calculated using Fisher’s exact test. *P < 0.05; **P < 0.01.

The effect of phage MS2 administration on plasmid loss from a *S*. Enteritidis from which the F-like virulence plasmid had been eliminated^30^ and into which F*lac*::Tn*3* was transferred, was studied during transmission of the strain through a group of young chickens. A standard seeder-bird experimental design was used in which a small number of birds within a group are infected, and transmission to in-contact (spreader) birds is monitored at intervals^34^. Although this tends to show transmission over a period of weeks in birds of more than few days old, it was expected that transmission between a small group of newly-hatched chickens would be very rapid when not treated with phage, as occurred (Fig. 2).

The results showed that MS2 controlled effectively the dissemination of the *S*. Enteritidis F*lac*::Tn*3* through the in-contact, spreader birds for two days post-infection with only 40% and 57% of the phage-treated birds infected on days 1 and 2 in comparison with 93% in the untreated birds at this time (P < 0.01, P < 0.05). However, by 4 days post-infection the infection levels were the same in both groups (Fig. 2). In addition, 8 birds from the control, untreated group required euthanasia due to the severity of disease; whereas none of the birds in the phage-treated group exhibited clinical signs of infection. The percentage of *S*. Enteritidis recovered from the birds, which also harboured the plasmid was statistically different between the phage-treated and control groups.

**Figure 3:**
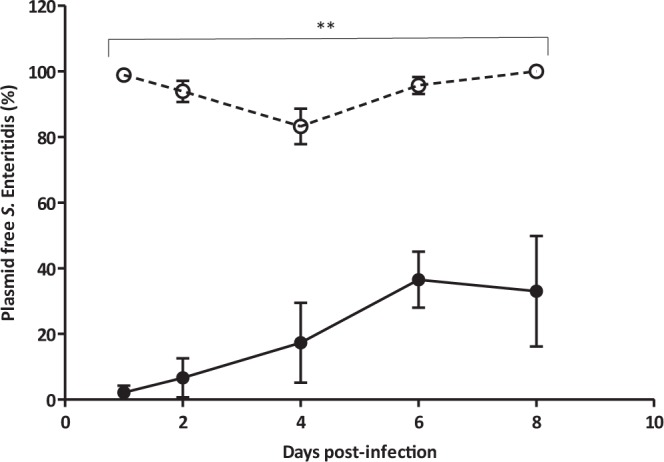
Plasmid-free presence of *S*. Enteritidis in seeder birds. Phage MS2 treated group (○) and control animals (•).Values represent mean proportion of plasmid free *S*. Enteritidis (n = 5) ± standard error. Significance was calculated using the Mann Whitney U test. **P < 0.01.

From day 1, the level of plasmid carriage in the phage-treated seeder birds was very low, with a proportion of plasmid-free colonies ranging between 83.2 ± 5.4% to 100 ± 0.0% during the experiment (P < 0.01) (Fig. 3). In contrast, the level of plasmid carriage in the control group was high throughout the experiment, although the frequency of plasmid-free bacteria increased from 2.1 ± 2.1% to 36.6 ± 8.6% between days 1 and 6 post-infection (Fig. 3). However, the presence of phage MS2 was associated with a significantly higher proportion of plasmid-free bacteria (P < 0.01). It is clear then that phage MS2 activity in the intestine accelerates this natural process.

**Figure 4:**
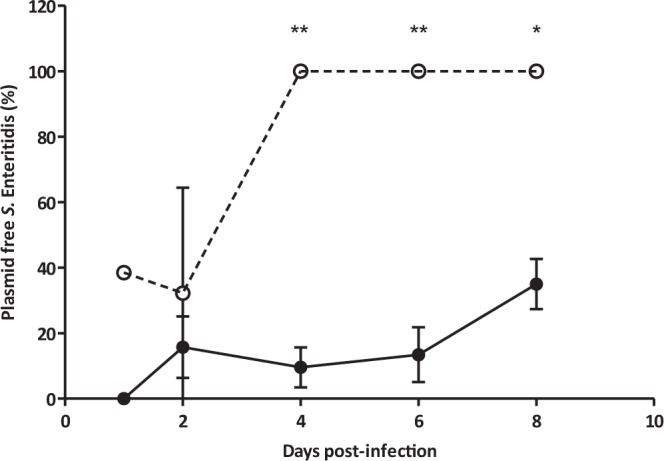
Plasmid-free population of *S*. Enteritidis in Spreader birds. Phage MS2 treated group (○) and control animals (•).Values represent mean proportion of plasmid free *S*. Enteritidis ± standard error (n = 10). Significance was calculated using Mann Whitney U test. **P < 0.01, *P < 0.05.

**Figure 5:**
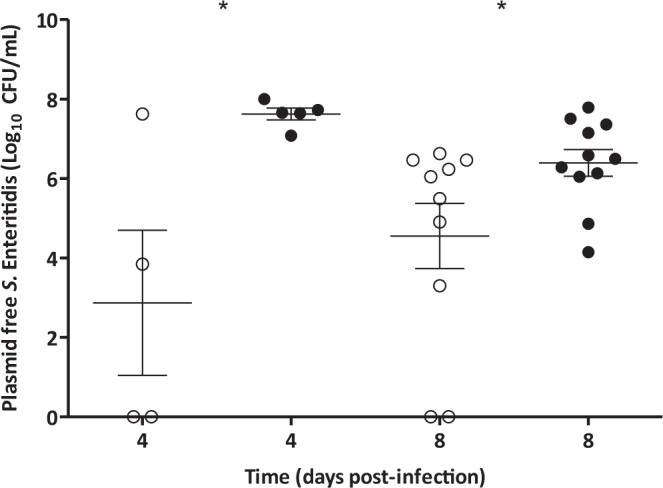
Plasmid-free *S*. Enteritidis in the caecum of Spreader birds. Phage MS2 treated group (○) and control animals (•), values represent concentration of plasmid free *S*. Enteritidis. The average for each group (—) ± standard error is also shown (n = 5, 10). Significance was calculated using Mann Whitney U test. *P < 0.05.

The results corresponded directly with the bacterial and plasmid population dynamics in the spreader birds. The proportion of plasmid free colonies was similar in both groups during the first two days of the experiment (Fig. 4). The low spread rate of *S*. Enteritidis in the phage-treated group reduced the difference in plasmid free *S*. Enteritidis between groups at this time. However, the situation changed completely after day 4 post-infection when *S*. Enteritidis spread was similar in both groups (Fig. 2). At that point, only plasmid-free *S*. Enteritidis was detected in the treatment group, while the proportion of plasmid-free bacteria reached a maximum of 35.0 ± 4.2% at day 8 post-infection in the control group (Fig. 4, P < 0.01). Similar results were observed for the populations of *S*. Enteritidis found in the caecum of the animals at days 4 and 8 post-infection (Fig. 5 P < 0.05). In the treated group, MS2 phage counts in the caeca were 6.9 ± 0.33 and 6.8 ± 0.13 Log_10_ PFU/mL at days 4 and 8 post-infection.

All plasmid-free derivatives were sensitive to ampicillin, and unlike in the *in vitro* studies, the few plasmid-carrying *S*. Enteritidis recovered from the treatment group remained sensitive to phage MS2. Therefore, the selective pressure exerted by the phage MS2 shifted the *Salmonella* population in the seeder birds to be plasmid-free. This new population spread through the flock, infecting the spreader birds and, perhaps as a result of greater metabolic fitness, overtook any plasmid-carrying bacteria resistant to MS2 phage.

### F-like plasmid characterization

The present and a previous study^35,36^ concentrated on plasmids which are relatively de-repressed and thus highly susceptible to sex pilus-specific phage. It was important to explore the effect of phage on wild-type plasmids which show varying degrees of repression for the expression of sex-pilus^32,37^.

The majority of AMR-plasmids isolated in recent years belong to Incompatibility group F^38,39^. The incompatibility group of the plasmids from a collection of wild-type AMR *E*. *coli* strains isolated from chickens^29^ was determined by PCR^40^. All the plasmids belonged to incompatibility group F (Table 2). Most AMR-plasmids are self-transmissible but at very variable rates both *in vitro* and *in vivo* although the latter is generally several orders of magnitude lower^41,42^. To have a better understanding of these plasmid dynamics, the presence of the repressor *finO* gene of the correct size was detected by PCR and correlated with the self-transmissibility rate calculated for all of them (Table 2).

**Table 2:** Plasmid transfer rate after conjugation with *E. coli* J62 Rif ^R^.

*E*. *coli* AMR-Plasmid	Genotype	Transfer rate (%)
pFlac::Tn*3*	F-like, *finO*^−^	95.7 ± 3.5
pF16	F-like, *finO*^+^	20.3 ± 0.7
pF18	F-like, *finO*^+^	80.2 ± 1.1
pF21	F-like, *finO*^+^	23.0 ± 0.9
pF26	F-like, *finO*^+^	99.5 ± 0.5
pF27	F-like, *finO*^+^	100 ± 0.0
pF28	F-like, *finO*^+^	96.8 ± 0.7

Values are presented as the mean of three replicates (n = 3) ± standard error.

As expected, the de-repressed plasmid F*lac*::Tn*3* had a very high transfer rate. In this plasmid, the repressor FinO is inactive due to an insertion sequence IS*3a* in *finO*^43^. This leads to a constitutive production of conjugative pili which promotes F-plasmid transmission. Despite the presence of an intact *finO* gene being detected in all the remaining F-like plasmids, four of them had almost the same transfer rates as F*lac*::Tn*3*. Only the plasmids pF16 and pF21 showed a degree of reduced conjugative ability. Therefore, the presence of an apparently functional FinO does not preclude relatively high transfer rates indicating a more complex control of sex- pilus expression and conjugation than expected.

### Phage MS2 host range

**Figure 6:**
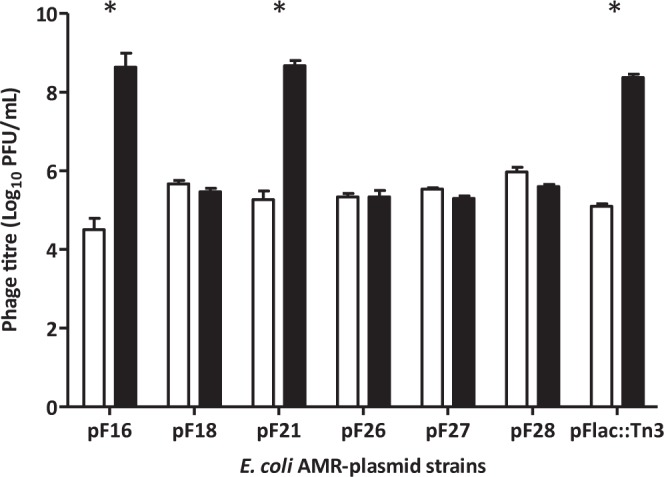
Phage MS2 replication in AMR-plasmid *E. coli* strains. White bars represent MS2 titre at time 0 h, while black bars show the titre after 24 h incubation at 37 °C with shacking. *E. coli* J62 Flac::Tn*3* was set as a positive control for phage MS2 replication. All values are the mean of three independent replicates ± standard error (n = 3). Significance was calculated using Mann Whitney U test. *P < 0.05.

We tested the ability of phage MS2 to infect the wild-type *E*. *coli* chicken strains carrying the semi-repressed AMR-plasmids which, of necessity, was tested in broth cultures because clear plaques would not form on bacterial lawns as a result of the low proportion of cells expression sex-pili (Fig. 5). This situation leads to a mixed bacterial population containing phage-resistant and phage-sensitive clones. In such conditions, phage MS2 plaques could not be observed on lawns of the host in top agar, because the resistant population would mask an developing plaques. Therefore, the only way to assess phage MS2 infectivity was to check the production of new virions in broth cultures of the strains. Figure 6 shows the initial and final phage concentration after 24 h incubation with the host strains.

Although all the plasmids were F-like, phage MS2 replication was observed only in strains carrying plasmids pF16 and pF21, with phage multiplication levels similar to those found in *E*. *coli* J62 F*lac*::Tn3 (Fig. 6). The reason is the presence of F sex-pilus which is a characteristic common to the IncF/MOBF12 plasmid group^44^. However, slight changes in phage MS2 receptor TraA protein, might explain the lack of infection in some of the strains isolated from chickens.

### Phylogenetic analysis based on *traA*

For the phylogenetic analysis, the nucleotide sequences of *traA* from the F-plasmids from this study were compared with the nucleotide sequences of *traA* from published F-plasmids from *E*. *coli* and *E*. *faecalis* (Fig. S1). The reference F-plasmid NC_002483.1 was used as positive control for phage MS2 infection. The phylogeny showed that the plasmids pF26 and pF28 were closely related and clustered together in clade 1. The reference F-plasmid NC_002483.1 was in clade 2, closer to the clade 3 which included pF21. The pF16 was most distantly related to pF26, pF28 and pF21 and was part of the clade 4. The multiple sequence comparison of the nucleotide sequence of *traA* showed that the reference published F-plasmid NC_002438.1 shared highest identity (99.73%) with the pF21 and was least identical to pF26 and pF28 (95.9%)(Fig. S2).

### The F-specific phage MS2 selects for loss of repressed F-like plasmids

**Figure 7:**
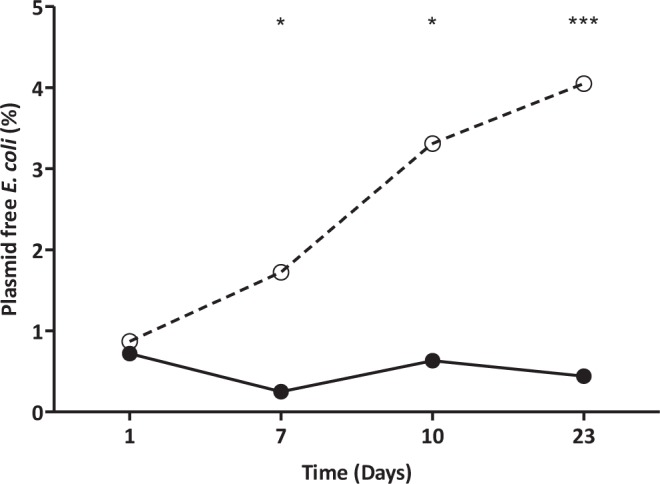
Phage MS2 effect on repressed plasmid pF21 presence over time. Phage MS2 treated group (○) and untreated control (•). Significance was calculated using Fisher’s exact test. *P < 0.05; **P < 0.01.

The effect of passage of *E*. *coli* pF21 in broth with the presence of MS2 phage was monitored over several days, with the result that selection for plasmid loss was also observed (Fig. 7). Although the plasmid loss increased from 0.9% to only 4.1% after 23 days in contact with MS2 phage, the reduction in plasmid population was ten times higher than in the untreated group (0.4%, P < 0.001). In a bacterial population carrying a more repressed plasmid, fewer bacterial cells will express sex-pili and be sensitive to MS2 phage.

### The F-specific phage MS2 blocks bacterial conjugation

To see whether the presence of phage MS2 was able to block plasmid transfer, phage was added during a conjugation *in vitro*. As expected, the phage was able to stop transmission of F*lac*::Tn*3*, reducing the transfer rate by 99.95% (Table 3). For the repressed plasmids pF16 and pF21, the effect of the phage was even greater as conjugation was completely undetectable.

**Table 3:** Plasmid transfer rate after conjugation with *E. coli* 711 Rif ^R^ with and without MS2 phage.

Plasmid	MS2 phage	Transfer rate (%)*	Reduction in transfer rate (%)
F*lac*::Tn*3*	—	95.7 ± 3.5	0
	MOI 10	0.05 ± 0.04	99.95
pF16	—	20.3 ± 0.7	0
	MOI 10	0 ± 0.0	100
pF21	—	23.0 ± 0.9	0
	MOI 10	0 ± 0.0	100

*Values are presented as the mean of three replicates (n = 3) ± standard error.

## Discussion

We have shown that lytic bacteriophages, which specifically use plasmid-associated sex pili as attachment points, not only have the potential to kill bacteria harbouring plasmids which are relatively highly de-repressed but also select for plasmid loss and block plasmid transmission. With a de-repressed plasmid, phage application led to an over 80% plasmid loss from a *S*. Enteritidis strain both *in vitro* and in the intestines in young chickens.

The effect of sex pilus-specific phages on plasmid stability, especially AMR-plasmids has been studied previously^35,36^. However, to the best of our knowledge this is the first time that this effect has been demonstrated *in vivo*. Previous reports suggested that a small number of phage of different specificities might be sufficient to cover the major replication types of AMR-plasmid^35^. Nevertheless, this might be over-optimistic as there are significant differences in sex-pilus structure even within the F-like plasmid group^45^. The *traA* relatedness of our collection of F-plasmids with the published F-plasmid sequences suggests that those published plasmids which grouped with the pF21, pF16 and NC_002438 represent a sex-pilus structure which phage MS2 will be able to infect. Therefore, phage MS2 would only infect conjugative F-plasmids carrying *traA* sequences closely related to clades 2, 3 and 4^44^. This suggests that a phage cocktail will be necessary to tackle different plasmids within the F-like plasmids.

The group of Jalasvuori has shown that phage PRD1, which attaches to the sex-pilus of IncP, N and W plasmids^46^, was able to select for plasmid loss^35,36^. As in that study, a small proportion of phage-resistant but nevertheless antibiotic resistant (plasmid-containing), strains arose as a result of the selective pressure of MS2 phage. The three F*lac*::Tn*3* derivatives analysed here were unable to conjugate and showed complete immunity to phage MS2 adsorption. Genomic analysis of MS2 phage-resistant mutant R4 revealed mutations in *traJ* region, which encodes the activator of the major transfer operon in the F-plasmid^47^. Thus, phage activity either eliminated the antibiotic resistance plasmid or eliminated the capacity to transmit the plasmid further. Phage MS2 induced extensive loss of the de-repressed F*lac*::Tn*3* *in vitro* and also a significant loss of the F-like pF21 plasmid from an AMR *E*. *coli* isolated from chickens. This effect was enhanced by extended contact of the plasmid carrying strains and MS2 phage, which was also observed for a phage PRD1 reported previously^36^. The lower rate of wild-type pF21 plasmid loss may be explained by its more repressed nature. In such repressed systems, where fewer bacterial population express sex-pili the effective MOI may be greater than for a completely de-repressed plasmid. However, plasmid transfer to a recipient population is epidemic with new recipients showing a burst of sex-pilus production resulting from momentary de-repression following low intracellularly concentrations of FinO and FinP^48^. Therefore, the presence of a sex pilus-specific phage in the environment at the time of conjugation activity could prevent plasmid transmission even if these are highly repressed. This hypothesis was confirmed, as MS2 phage completely blocked transmission of pF16 and pF21. Similar findings were obtained with the de-repressed RP4 plasmid^35,36^. This suggests that sex pilus-specific phage administration might reduce epidemic spread of de-repressed and repressed plasmids.

Although the results suggest that phage administration might reduce also plasmid spread, this approach certainly shows the effect of SPS phage on plasmid carriage during normal bacterial growth, both *in vitro* and *in vivo*.

We studied the effect of MS2 phage administration on the carriage and spread of a *Salmonella* containing the de-repressed F*lac*::Tn*3* in a chicken flock. The *in vivo* trial showed that in the intestines of very young chickens, where *Salmonella* may colonise nearly all regions of the gut^49^, the administration of phage MS2 reduced the transmission of the *Salmonella* with the plasmid between birds by direct reduction in the numbers of plasmid-carrying bacteria. There was notable plasmid loss in both the birds which were inoculated directly (seeder) and in those where the infection occurred by contact with the directly infected birds (spreaders). Interestingly, a progressive loss of plasmid in the untreated animals occurred, although this was much lower than in the phage-treated birds. This natural plasmid loss is likely to be a consequence of the high metabolic cost of a constitutive expression of the transfer apparatus^48^ in the absence of any evolutionary pressure selecting for maintenance of the plasmid. In contrast to the results obtained *in vitro*, no phage-resistant *Salmonella* mutants were recovered which nevertheless continued to carry the plasmid. It is possible that the metabolic burden of the 100 kb F-plasmid^50^ made very difficult the competition of the plasmid-containing, phage-resistant mutants against the plasmid-free *Salmonella*, which overtook and colonized the gut much faster.

These results indicate that the use of SPS phage has a three-fold advantage against AMR bacteria where the resistance is plasmid-mediated, namely (i) the killing effect in environments *in vitro* and *in vivo* where good contact can be made between phage and sex-pilus, particularly where *in vivo* gut contents are fairly liquid; (ii) the selection of plasmid-free derivatives which could outgrow the remaining phage-resistant AMR mutants; (iii) the reduced ability of the phage-resistant AMR mutants to self-transmit their plasmids.

This group envisages that sex pilus-specific phages might be applied under two sets of conditions, either in a population (animal or human) or in an individual. In the former situation the present results suggest that, in the absence of antibiotic administration and pressure, widespread phage use should drive bacterial evolution towards plasmid and AMR loss as the bacteria multiply in and spread between individuals. Thus, despite continuing concerns over phage application in the environment where phage-resistance could develop this is one situation where it does not appear to be a major concern since the phage-resistant derivatives are desirable. In individual treatment, such as a septicaemia case, bacterial numbers may be reduced by treatment with sex-pilus specific phage such that the host’s immunity may be able to deal with it, as suggested previously^20,21^. The advantage conferred by plasmid loss should result in the gradual replacement by antibiotic sensitive bacterial derivative cells, which if transmitted to other individuals should proliferate at the expense of AMR strains.

## Methods and Materials

### Bacterial strains, culture media

*E*. *coli* J62 *lac pro trp his* pF*lac*::Tn*3* (Amp^R^) and *Salmonella* Enteritidis P125109^51^ from which the virulence plasmid had been eliminated^52^ and the pF*lac*::Tn*3* introduced (Amp^R^) were used as host for phage MS2. A rifampicin resistant mutant of the *Salmonella* strain (Amp^R^, Rif ^R^) was used for the *in vivo* experiments. A collection of plasmid-containing wild-type AMR *E*. *coli* strains isolated from poultry^37^ were selected for host range, phage MS2 sensitivity and plasmid loss experiments (Table S1). *E*. *coli* J62 Rif ^R^ was used as recipient strain for plasmid transfer rate assessment.

Luria Bertani (LB) broth and agar (1%, Sigma) was used for routine culture of the strains. Viable counts of *E*. *coli* J62 pF*lac*::Tn3 (Amp^R^) and AMR strains were determined by plating dilutions on MacConkey agar (Sigma) with or without antibiotics. The same was done for *S*. Enteritidis P125109 pF*lac*::Tn3 (Amp^R^, Rif ^R^) counts, using Brilliant green agar (Sigma) supplemented with the appropriate antibiotic. For dilution purposes Phosphate-Buffered saline (PBS) buffer was used.

### Plasmid incompatibility group determination

The presence of F-like plasmids was determined by PCR following a previously described method^40^ with slight modifications. A set of primers were used to recognise four specific regions of replicons that are representative of plasmids belonging to the Incompatibility F group (Table S2). The multiplex and the singleplex PCR cycling conditions consisted of an annealing temperature of 60 °C and 52 °C for 30 sec respectively. For both reactions, the initial denaturation step was performed at 94 °C for 5 min, denaturation at 94 °C for 1 min and an elongation and final extension at 72 °C at 1 min and 5 min respectively. The presence of the repressor *finO* was also detected by PCR. For that, primers were designed using Primer3 based on the genomic sequence NC_002128.1 (Table S2). Similar cycling conditions as for the multiplex PCR were used except that the extension time was run for 40 seconds. Amplicons were then analysed on 1% agarose gel.

### Bacteriophage and propagation

Liquid lysates of phage MS2^30^ and phage EA (10 ml) were prepared by inoculating mid-exponential cultures of the plasmid-carrying strain *E. coli* J62 at a multiplicity of infection (MOI) of 0.1. The mixture was then incubated overnight at 37 °C with shaking at 150 rpm. After incubation, the lysate was centrifuged at 10,000 × g for 10 min and filtered through a 0.45 μm syringe filter (Sartorius). In all cases, phage titres were determined by spotting 10 µl of decimal dilutions onto LB plates using the double agar layer method^53^.

Double agar layer method^53^.

### Phage adsorption

An *E. coli* J62 pF*lac*::Tn*3* culture at mid-exponential phase was infected with phage MS2 at an MOI of 0.1. The adsorption rate was determined by taking samples every 2 min for up to 14 min. At each time point, adsorbed phage were removed by centrifugation at 13,000 × *g*. After this, the supernatant was filtered using 0.45 μm filter (Millipore) and unbound phage were plated on LB agar overlays containing 0.1 mL of the host strain. The plates were then incubated overnight at 37 °C before enumeration.

### MS2 host range

Ten millilitre LB broth aliquots were inoculated with 100 µl of the relevant wild-type AMR *E*. *coli* strain or *E*. *coli* J62 pF*lac*::Tn*3* overnight cultures. The mixtures were then incubated 2–3 h at 37 °C with shaking until the mid-exponential phase was reached. At that point, MS2 phage was inoculated at a final concentration of 10^5^ PFU/mL, giving a final MOI of 0.001. One millilitre samples were taken at time 0 and 24 h and filtered through 0.45 µm syringe filters to check phage titre^53^. Significant increases between initial (0 h) and final (24 h) phage titre indicated phage replication in the host strain.

### Effect of MS2 phage during conjugation

Overnight LB broth cultures of the recipient *E*. *coli* J62 Rif^R^ and donor wild-type AMR *E*. *coli* strains and *E*. *coli* J62 pF*lac*::Tn*3* were prepared. For the conjugation 5 µL of both donor and recipient strain were diluted in the same 5 mL LB broth aliquot with or without MS2 phage at a final MOI of 10. The mixture was then incubated overnight at 37 °C without shaking. After incubation, dilutions were plated on MacConkey agar supplemented with rifampicin (100 µg/mL) to enumerate the recipient strain. Similarly, for the trans-conjugant counts dilutions were plated on MacConkey agar rifampicin (75 ug/mL) and an appropriate antibiotic for the donor plasmid. The plasmid transfer rate was calculated as:

$$\frac{{\rm{Trans}}-{\rm{conjugant}}(\frac{{\rm{CFU}}}{{\rm{mL}}})}{{\rm{Recepient}}\,(\frac{{\rm{CFU}}}{{\rm{mL}}})}$$

### Plasmid population kinetics with the F plasmid

An overnight LB broth culture of *E*. *coli* J62 pF*lac*::Tn*3* was diluted 1/100 in two 10 mL aliquots of LB broth. The fresh cultures were incubated for 2 h at 37 °C with shaking. After incubation, one of the aliquots was inoculated with 100 µL of a MS2 phage preparation to give a final MOI of 10. Both cultures were then incubated with shaking for 24 h at 37 °C. The cultures were again diluted 1/100 in fresh LB broth and 100 *μ*L of MS2 phage preparation was added to treatment group as before. This process was repeated again at 48 and 72 h. Samples were taken each 24 h and plasmid loss was studied. For this, serial dilutions were plated on MacConkey agar and 100 colonies were replicate-plated using MacConkey agar without and with ampicillin (100 µg/mL). The percentage of plasmid loss was then calculated as:

$$\,(\frac{{Ampicillin}\,{sensitive}\,{colonies}}{{Total}\,{number}\,{of}\,{colonies}})\times 100.$$

### Plasmid population kinetics with a F-like repressed plasmid

An overnight LB broth culture of the *E*. *coli* strain carrying the pF21 plasmid (Sm^R^) was diluted 1/100 in two 10 ml volumes of LB broth. To one of those, a 100 µL of MS2 phage preparation was inoculated to give a final MOI of 10. Both cultures were incubated with shaking for 24 h at 37 °C and were then diluted 1/100 in fresh LB broth adding 100 µL MS2 preparation as before. This process was continued daily for 23 days. At 0, 1, 7, 10 and 23 days after incubation a diluted aliquot of each culture was plated on 5 MacConkey plates, aiming for a count of 200 well separated colonies on each plate. These plates were incubated overnight and replica-plated to MacConkey plates with or without streptomycin (100 µg/ml)to which the strain was resistant. After incubation colonies which had lost the antibiotic resistance were identified and plasmid loss rate was calculated as mentioned above.

### Characterization of AMR-plasmid isolates resistant to MS2 phage

*E*. *coli* J62 pF*lac*::Tn*3* colonies which were phage-resistant after MS2 phage treatment were characterized. The presence of plasmid was tested by PCR with F-like plasmid specific primers^40^. Resistance to ampicillin was also tested by plating on MacConkey agar supplemented with ampicillin (100 µg/mL). Phage MS2 adsorption was performed as described previously^54^ and studies of plasmid transfer rate were done as described above.

### Genomic and phylogenetic analysis based on *traA*

Raw sequence reads generated as FastQ file for each F-plasmid were assembled *de novo* using SPAdes (v3.6.2)^55^. The contigs with size more than 1000 bases were extracted and further analysed. The nucleotide sequence of *traA* from the reference published F-plasmid from *E*. *coli* K-12, NC_002438.1 was used as a query in BLAST+^56^ against the genome sequences of the F-plasmids. The nucleotide sequences of *traA* from the corresponding F-plasmids were extracted using the start and end positions from the BLAST output. The nucleotide alignments were produced by ClustalW (CLUSTAL 2.1)^57^. The maximum likelihood phylogenetic analysis was performed using the generalized time-reversible (GTR) model to infer nucleotide evolution with FastTree^58^. The phylogeny was visualised using FigTree (http://tree.bio.ed.ac.uk/software/figtree/).

### Chicken experiment

Chicken experiments were designed following the ARRIVE guidelines (https://www.nc3rs.org.uk/arrive-guidelines) and approved by the University of Nottingham Animal Welfare and Ethical Review Body (Approval Non UK 0006 Phage-mediated loss of antimicrobial resistance) and by the FCAV, UNESP ethical review panel. All the experiments were performed in accordance with the rules set by the University of Nottingham Animal Welfare and Ethical Review Body and by the FCAV, UNESP ethical review panel.

Two groups of 15 newly hatched chicks were housed separately in cages (95 × 95 × 30 cm) and provided with chicken mash and water *ad libitum*. They were placed in clean rooms at ambient temperature and provided with heat lamps. All birds were swabbed on receipt to check for freedom from *Salmonella*. The swabs were incubated in selenite broth and plated on to plain and rifampicin supplemented (100 µg/mL) Brilliant green agar.

Two groups of 15 newly hatched chickens (Ross 308) were infected with *S*. Enteritidis P125109 pF*lac*::Tn*3* (Amp^R^ Rif ^R^). One group (Treatment) was treated with MS2 phage, while the other control group was untreated. The birds were kept for 8 days and housed in separated rooms with appropriate precautions taken to prevent cross infection between rooms. All the inoculations were oral and performed using blunt feeding needles.

For the inoculations 5 birds in each group were marked with red fuchsin and inoculated at day 1 with 0.2 mL of an overnight culture of the *S*. Enteritidis pF*lac*::Tn*3* Amp^R^ Rif ^R^ strain. Immediately after the bacterial inoculation the treatment group were inoculated orally with 0.1 mL of antacid solution (10% CaCO_3_) followed by 0.1 mL of phage MS2 suspension at 10^11^ PFU/mL. Each bird in the control group was dosed with 0.2 mL of an antacid preparation only. Phage and antacid inoculation was repeated every day during the experiment. The water in the treatment group was also supplemented with MS2 phage at a final concentration of 10^9^ PFU/mL.

On days 1, 2, 4, 6 and 8 post-infection all birds were cloacal-swabbed. Swabs were taken before each phage inoculation and placed in tubes containing 2 mL PBS. After brief mixing on a vortex mixer each swab was plated in a standard manner^59^ on a half Brilliant Green agar plate supplemented with rifampicin (100 µg/mL). The plates were incubated overnight at 37 °C and scored for *Salmonella* presence as previously reported^59^. At day 4 post-infection 5 birds were sacrificed from each group and the caecal contents processed to enumerate the inoculated *S*. Enteritidis Rif ^R^. This was repeated at day 8 with the remaining birds from both groups. Decimal dilutions of caecal contents were plated on Brilliant Green agar plate supplemented with rifampicin (100 µg/mL). The numbers of lactose-positive, presumed plasmid-positive (Green) and lactose-negative, presumed plasmid-negative (Red) colonies were checked for each animal for both cloacal and caecal samples. To confirm antibiotic sensitivity 20 colonies of both colour types were plated onto Brilliant Green agar supplemented with rifampicin and ampicillin at a 100 µg/mL. Those 40 colonies were also tested for phage susceptibility by spotting 10 µL of MS2 phage (10^9^ PFU/mL) onto lawns. Phage counts in caecal contents were determined on days 4 and 8 post-infection. For that, the initial x10 caecal dilutions were heated at 58 °C for 30 min to kill the bacteria. Further decimal dilutions were made and 10 µL aliquots were plated on lawns of *S*. Enteritidis P125109 pFlac::Tn*3* (Amp^R^ Rif ^R^) growing on rifampicin LB agar (100 µg/ml).

### Statistical analysis

Phage MS2 induced plasmid loss *in vitro* and *Salmonella* spread through the chicken flock were analysed using Fisher’s exact test (Graphpad Prism, version 7.01). To evaluate the differences in plasmid presence and phage production, all bacterial and phage counts data were Log_10_-transformed prior to statistical analysis. For data not following a normal distribution according to the Shapiro-Wilks normality test a Mann Whitney U test was done. (SPSS statistics 24 software, IBM). A two way ANNOVA with Bonferroni post-test was performed for samples following a normal distribution.

## Supplementary information

Figure S1

## Data Availability

The authors declare that the data supporting these findings is available within the paper or in its supplementary material.
