# Supplementary material for: Sex pilus specific bacteriophage to drive bacterial population towards antibiotic sensitivity
Source: Sci Rep. 2019 Aug 30;9:12616. doi: 10.1038/s41598-019-48483-9 (PMC6717204; doi:10.1038/s41598-019-48483-9)
Supplement: Supplementary file 1 — Figure S1 [file 41598_2019_48483_MOESM1_ESM.pdf]

Sex pilus specific bacteriophage to drive bacterial population towards antibiotic sensitivity.

Joan Colom[1], Diego Batista[2], Abiyad Baig[1], Ying Tang[1], Siyang Liu[1], Fangzhong Yuan[1], Aouatif Belkhiri[1], Lucas Marcelino[2], Fernanda Barbosa[2], Marcela Rubio[2], Robert Atterbury[1], Angelo Berchieri[2] and Paul Barrow[1*].

[1]School of Veterinary Medicine and Science, University of Nottingham, Sutton Bonington, Leicestershire, LE12 5RD UK.

[2]School of Agricultural and Veterinary Sciences, São Paulo State University (FCAV/UNESP), São Paulo, 14884-900, Brazil.

* Corresponding author

E-mail: Paul.barrow@nottingham.ac.uk

Phone: +44 115 951 6428

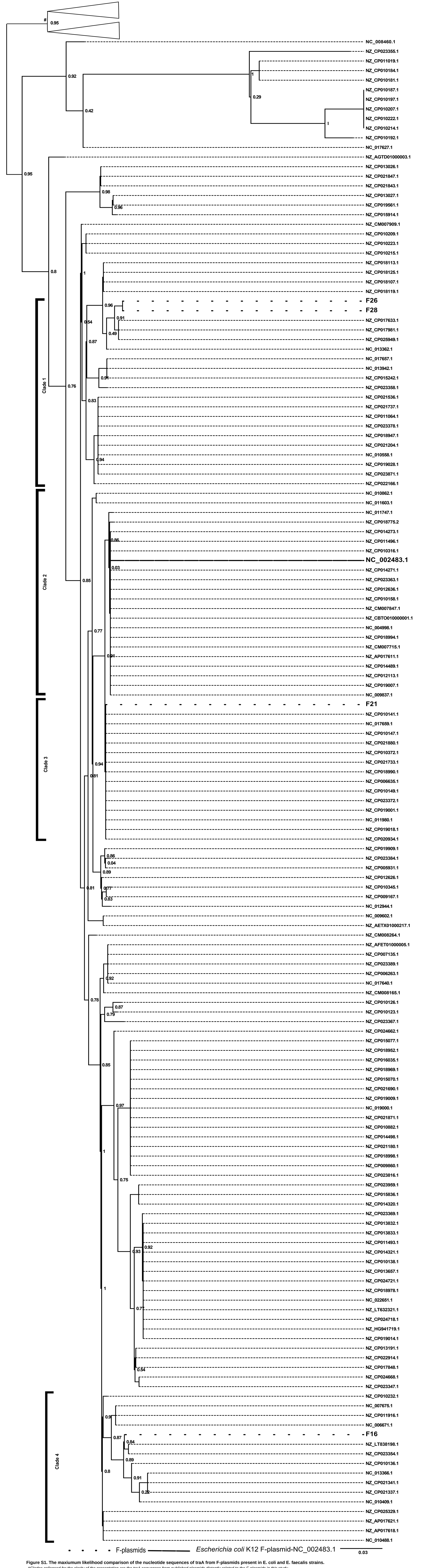

Figure S1. The maxiumum likelihood comparison of the nucleotide sequences of traA from F-plasmids present in E. coli and E. faecalis strains.

#Clades collapsed for the clarity of the presentation are the traA sequences from published plasmids distantly related to the F-plasmids in this study.
